# Incidental Finding of Intracavitary Right Coronary Artery in an Adult Patient with Tetralogy of Fallot: A Case Report

**DOI:** 10.7759/cureus.77914

**Published:** 2025-01-24

**Authors:** Ahmed Alhejji, Ali Alsehaiw, Ali Abdullah Alsharit, Abdulraouf Mohammed Alobaid, Fatima M Almohammed saleh

**Affiliations:** 1 Radiology, Qatif Central Hospital, Qatif, SAU; 2 Radiology, Alahsa Health Cluster, Hofuf, SAU; 3 Emergency Medicine, Alahsa Health Cluster, Hofuf, SAU; 4 Internal Medicine, King Fahad Hospital Hufof, Hofuf, SAU; 5 College of Medicine, King Faisal University, Hofuf, SAU

**Keywords:** congenital heart disease, coronary artery anomalies, intraarterial course of right coronary artery, intracavitary right coronary artery, tetralogy of fallot

## Abstract

Tetralogy of Fallot (TOF) is a cyanotic congenital heart defect comprising stenosis or atresia of the pulmonary valve, ventricular septal defect, overriding aorta, and right ventricular hypertrophy. TOF is known to be associated with coronary artery anomalies. We describe a rare case of TOF associated with an intracavitary right coronary artery (RCA) course. The recognition of such an anomaly is vital before surgical/interventional procedures to avoid catastrophic outcomes.

## Introduction

Tetralogy of Fallot (TOF) is a cyanotic congenital heart condition comprising four heart defects: stenosis or atresia of the pulmonary valve, ventricular septal defect, overriding aorta, and right ventricular hypertrophy. TOF is the most common cyanotic heart disease. The prevalence of coronary artery anomalies is known to be higher in patients with TOF than in the general population [1].

The possible coronary artery anomalies can be classified as anomalies of origin, anomalies of course, and anomalies of termination [2]. When a portion of the right coronary artery (RCA) passes through the right atrial chamber, it is known as the intra-atrial or intra-cavitary course of the RCA. It is an uncommon vascular malformation, with an incidence of around 1% [3].

In this report, we describe a rare case of TOF associated with an intracavitary RCA course.

## Case presentation

A 28-year-old male, with known intellectual disability, TOF, and patent foramen ovale (PFO) since birth, status post TOF complete repair and PFO closure in childhood at the age of 20 months, complicated by severe pulmonary regurgitation, presented to the cardiology clinic for follow-up after a recent history of ischemic stroke to rule out a cardioembolic source. The patient reported longstanding exertional shortness of breath after moderate activity which interfered with his daily activity. On physical examination, the patient looked well, not distressed or cyanosed. The body temperature was 36.9° C, blood pressure 120/71 mmHg, heart rate 65 beats/minute, oxygen saturation 98%, and respiratory rate 18 breaths/minute. The chest examination revealed an anterior median sternotomy scar, and clear lungs without added sounds. The heart examination revealed an early diastolic murmur grade 3/6. The abdomen was soft without organomegaly, and the lower limbs revealed no pitting edema. Electrocardiography (ECG) showed normal sinus rhythm and echocardiography showed mild tricuspid valve insufficiency, severe pulmonic valve insufficiency, no pericardial effusion, and mildly decreased left ventricular systolic function.

**Figure 1:**
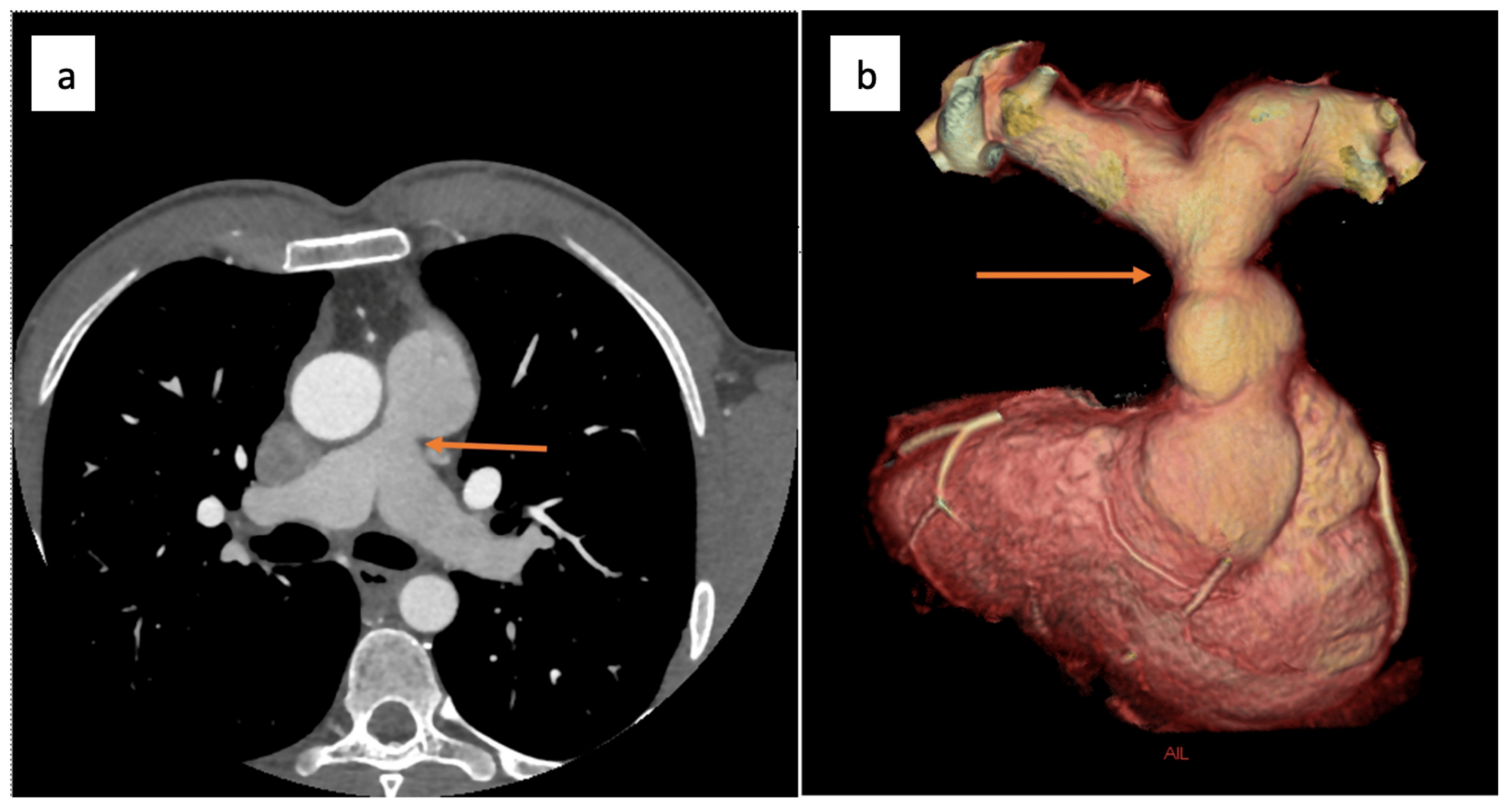
(a) Axial cardiac CT and (b) 3D volume rendering images of main pulmonary artery and side branch pulmonary arteries reveal focal supravalvular pulmonary artery stenosis (arrows) 3D: three-dimensional

**Figure 2:**
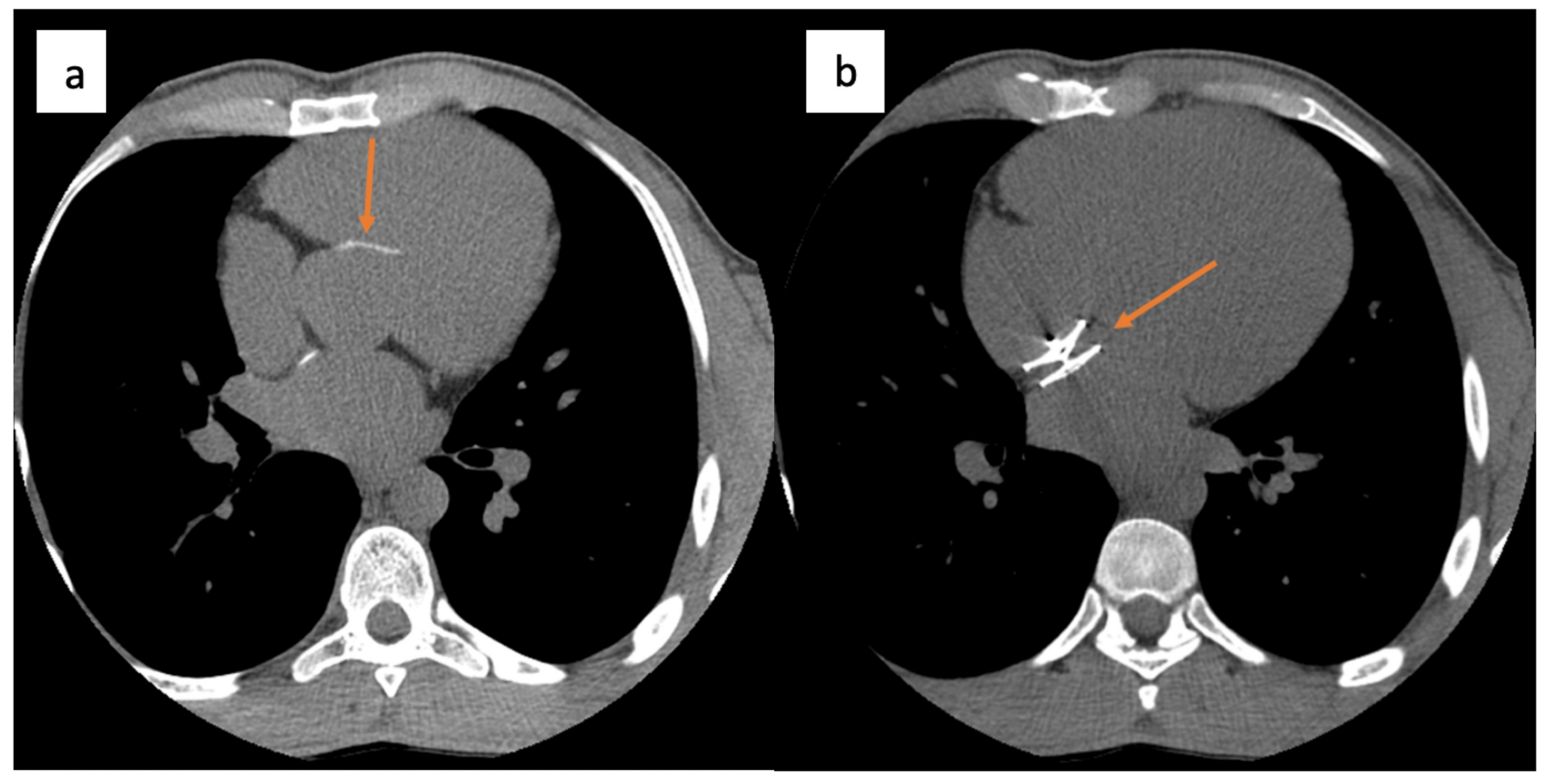
Non-enhanced prospective ECG-gated cardiac CT scan showing (a) the presence of a subaortic membranous ventricular septal defect patch (arrow) and (b) PFO occluder device “arrowed”. PFO: patent foramen ovale

**Figure 3:**
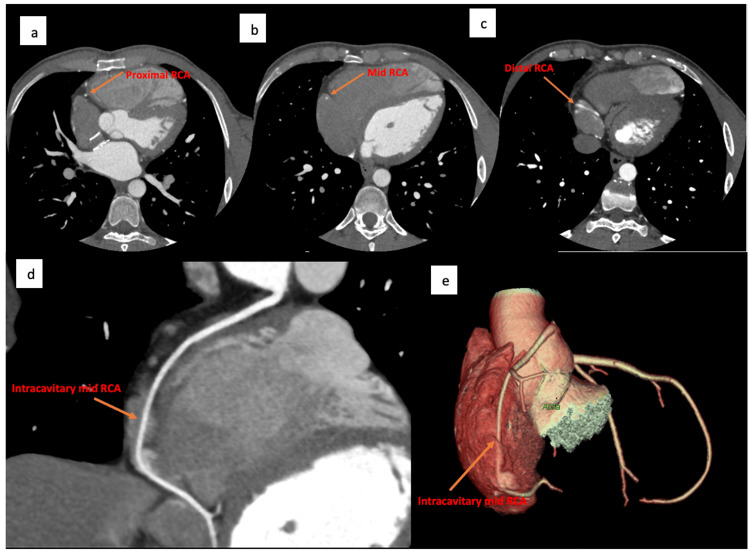
Axial, curved multiplanar reformatted as well as 3D volume rendering images from CT coronary angiogram demonstrate (a) Normal course of the proximal RCA segment along the right atrioventricular groove surrounded with epicardial fat, and (b, c, d, e) Intracavitary course of the mid and distal segments of the RCA within the right atrium. RCA: right coronary artery

**Figure 4:**
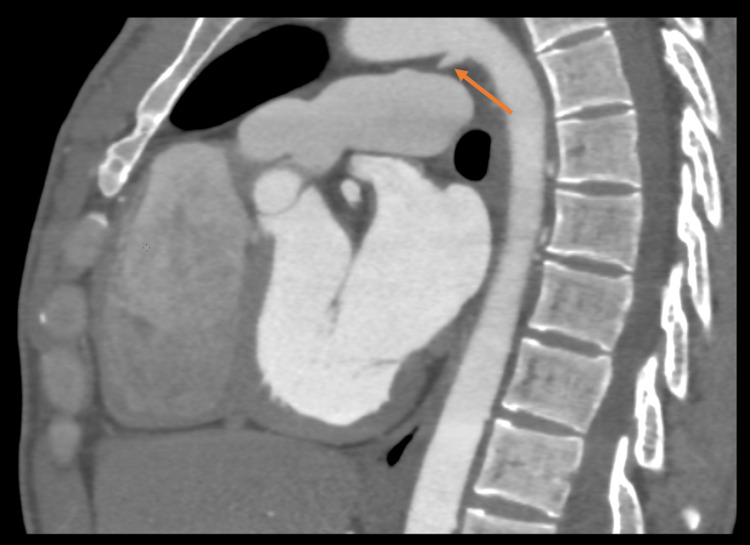
Sagittal image of coronary CT angiogram showing incidental conical-shaped patent ductus PDA (arrow) PDA: patent ductus arteriosus

A cardiac computed tomography (CT) scan was ordered to assess the coronary arteries, right ventricular outflow tract (RVOT), pulmonary artery, pulmonary veins, and pulmonary valve. A multiphasic retrospective ECG-gated coronary CT angiogram was obtained, which showed mild supraventricular main pulmonary artery (MPA) focal stenosis with ﻿dilated RVOT (Figure 1), intraventricular septal patch, and PFO occluder device (Figure 2). An incidental finding showed an intracavitary RCA (Figure 3) and conical-shaped patent ductus arteriosus (PDA) (Figure 4). The patient was referred to a cardiac center for further assessment and management.

## Discussion

TOF is the most common cyanotic congenital heart disease, with a prevalence of 5-10% of all congenital heart diseases and an incidence of three in 10,000 births [1,4]. A meta-analysis showed that about 6% of patients with TOF have anomalous coronary arteries and 72% cross the RVOT, with the remaining 28% course behind the aorta. Six percent of patients have a large conus artery and 4% have a coronary arteriovenous fistula. The left coronary artery or RCA from the pulmonary trunk or the left or right pulmonary artery, coronary tree hypoplasia, and anastomoses between the coronary and the bronchial artery are other coronary anomalies [5].

The intracavitary RCA is a rare anatomical variant with an incidence of about 0.09-0.1% in the general population [3]. The coincidence of TOF and intracavitary RCA is extremely rare with a prevalence of less than 1% of TOF patients [1]. Intracavitary RCA is considered one of the most malignant courses that can lead to hazardous events during interventional or surgical procedures [3]. A recent publication reported two cases of TOF with intra-atrial course of a coronary artery [1]. One case involved the left anterior descending coronary artery arising from the RCA and the other involved the RCA originating from the left main coronary artery. 

Multi-detector computed tomography angiography (MDCTA) is the gold standard and non-invasive diagnostic modality for assessing coronary artery anomalies, which provides detailed anatomical images that suggest high-risk coronary artery anomalies [6,7]. Coronary magnetic resonance has been introduced as an alternative to MDCTA in patients with coronary artery anomalies. In addition to offering similar anatomic details of the origin and course of the coronary arteries, it also provides valvular and systolic function, as well as assessing the myocardium. However, due to lower spatial resolution, the unavailability of expertise, as well as the specific instrumentation, coronary magnetic resonance has been only described as a secondary tool for assessing coronary artery anomalies [8,9].

## Conclusions

This report described a case of TOF associated with incidental findings of an intracavitary RCA and conical-shaped PDA. The recognition of such an anomaly is vital before surgical/interventional procedures to avoid catastrophic outcomes.
